# Observational Research, Randomised Trials, and Two Views of Medical Science

**DOI:** 10.1371/journal.pmed.0050067

**Published:** 2008-03-11

**Authors:** Jan P Vandenbroucke

## Abstract

Two views exist of medical science, says the author, one emphasizing discovery and explanation, the other emphasizing evaluation of interventions.

**Summary**

Two views exist of medical science: one emphasises discovery and explanation, the other emphasises evaluation of interventions. This essay analyses in what respects these views differ, and how they lead to opposite research hierarchies, with randomisation on top for evaluation and at bottom for discovery and explanation. The two views also differ strongly in their thinking about the role of prior specification of a research hypothesis. Hence, the essay explores the controversies surrounding subgroup analyses and multiplicity of analyses in observational research. This exploration leads to a rethinking of the universally accepted hierarchy of strength of study designs, which has the randomised trial on top: this hierarchy may be confounded by the prior odds of the research hypothesis. Finally, the strong opinions that are sometimes displayed in pitting the two types of medical science against each other may be explained by a difference in “loss function”: the difference in penalty for being wrong. A longer, more detailed version of this paper is found in supplementary Text S1.

Two views about medical science seem to have split ever more apart over the past decades. One view is that of medical researchers who rejoice in discoveries and explanations of causes of disease. Discoveries happen when things are suddenly seen in another light: the odd course of a disease in a patient, the strange results of a lab experiment, a peculiar subgroup in the analysis of data, or some juxtaposition of papers in the literature. Researchers get enthusiastic about an idea, and try to find data—preferably existing data—to see whether there is “something in it”. As soon as there is a hint of confirmation, a paper is submitted. The next wave of researchers immediately tries to check this idea, using their own existing data or their trusted lab experiments. They will look at different subgroups of diseased persons, vary the definition of exposures, take potential bias and confounding into account, or vary the lab conditions, in attempts to explain why the new idea holds—or why it is patently wrong. In turn, they swiftly submit their results for publication. These early exchanges may lead to strong confirmation or strong negation. If not, new studies are needed to bring a controversy to resolution.

The other view is that of medical researchers whose aim is to set up studies to evaluate whether the patient's lot is really improved by the new therapies or diagnostics that looked so wonderful initially. The most developed branch of evaluation research is randomised trials of drug therapy. One major condition for credibility of such trials is complete preplanning of every aspect of the trial, and nowadays even advance registration and documentation of everything that was preplanned [1]. This preplanning should not be strayed from, however promising some side alley looks, because the credibility of the results will immediately take a nose dive.

## What they think about each other.

From the perspective of the evaluative researcher, this method of discovery and explanation is dangerously biased: clinicians present case series out of the blue, epidemiologists do multiple analyses on existing data collected for completely different purposes, basic scientists repeat lab experiments with endless new variations, changing the hypothesis as well as the experiment continuously—until something fits. And all these researchers always dream up perfect explanations. This leads to irresponsible “hypes” and “scares” in the popular press, and to unnecessary research loops that are a burden to the public purse.

In contrast, the discovery type of researcher is convinced that too much emphasis on evaluation actually hampers the progress of science—precisely because everything is preplanned. For discovery you need chance and one-sided views. You need to look at the literature in a slanted way, to examine the data of others as well as your own to see them in a different light. To discoverers, evaluation is mainly a form of “quality control” that society needs for financial reimbursement by third party payers. Finally, numbers are not explanations; they do not give insight upon which you can build the next step of your reasoning or your next investigation.

## Co-existence in the mind of an individual?

Yet, these two views of medical science can exist simultaneously in the mind of one person. Over the past decades, I may have made one contribution to unravelling the aetiology of a disease: the detection of the interaction between factor V Leiden and oral contraceptives in causing venous thrombosis [2]. Young women who carry the factor V Leiden mutation (about 5% of the population of white European descent) and also use oral contraceptives have a much higher risk of venous thrombosis than women with either risk factor alone (Table 1).

**Table 1:**
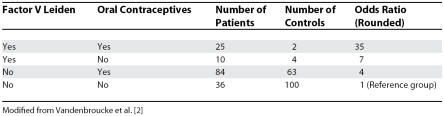
Analysis of Oral Contraceptive Use, Presence of Factor V Leiden Allele, and Risk for Venous Thromboembolism

This finding was not preplanned. Our study originally aimed at quantifying existing biochemical and genetic risk factors for venous thrombosis. The factor V Leiden mutation, a new risk factor for venous thrombosis, was discovered in part through data from the study [3]. After the mutation was established, we looked at the data again. We found a few homozygotes for the mutation among the patients. To our surprise, they were almost all young women who used oral contraceptives. We felt this might be the beginning of an explanation of why oral contraceptives cause venous thrombosis, and analysed homozygotes and heterozygotes together for the interaction with factor V Leiden. The findings indeed provided insight into the question of why exogenous hormones cause venous thrombosis [4].

However, whenever I suspect that a report from a randomised controlled trial has strayed from the path of complete preplanning, e.g., by cutting corners in the follow-up or emphasising some subgroup, I might be the first to cry “beware” [5]. While the two views on medical research lead to completely different mindsets about subgroups and exploring new findings in data, I do teach and encourage both to young researchers.

## Different Hierarchies for Different Problems

Underlying these differences in views are differences in the hierarchy of research designs that apply to different problems. A hierarchy of “strength” of research designs with the randomised trial on top and the anecdotal case report at a suspect bottom has been well known since the 1980s in various guises [6] and under various names. A typical rendering is shown in Box 1. I have qualified this hierarchy by naming it the hierarchy of study designs for “intended effects of therapy”, i.e., the beneficial effects of treatments that are hoped for at the start of a study.

**Box 1. Hierarchy of Study Designs for Intended Effects of Therapy**

1. Randomised controlled trials
2. Prospective follow-up studies
3. Retrospective follow-up studies
4. Case-control studies
5. Anecdotal: case report and series

The opposing hierarchy ranks study designs in the order in which they give the best chances of discovery and of studying new explanations, and is shown in Box 2. The entries in the second hierarchy are almost the same, except that the ranking is reversed. The first entry is somewhat enlarged, as anecdotal reports that lead to new ideas comprise not only case descriptions of patients, but may have other sources, e.g., data and literature. Any clinician or laboratory researcher will immediately recognise that this is how new discoveries are made. Odd observations in patients, data, or the literature spark a new idea, and only thereafter do analytic designs come into play.

**Box 2. Hierarchy of Study Designs for Discovery and Explanation**

1. Anecdotal: case reports and series, findings in data, literature
2. Case-control studies
3. Retrospective follow-up studies
4. Prospective follow-up studies
5. Randomised controlled trials

### A juxtaposition of the hierarchies.

In both hierarchies, there are large gaps of credibility and usefulness between the different levels. For evaluation of intended effects of therapy, the randomised controlled trial stands out, followed at quite a distance by all observational designs. Observational studies of intended effects of therapy suffer from nearly intractable problems of “confounding by indication”. Only very rarely will we believe case reports or series as evidence for therapy, for instance when effects are dramatic [7,8].

For discoveries, the original case reports, lab observations, data analysis, or juxtaposition of ideas in the literature may be so convincing that they stand by themselves [7,8]. In most instances, however, we need other studies to see whether the observation holds. The preferred designs of researchers are case-control studies, or possibly retrospective follow-up studies, because these designs will give the quickest answer for the least effort, and no further evidence may be needed. If at all possible, researchers will use existing data. A truly prospective follow-up study (i.e., involving new data collection and start of follow-up after the formulation of a specific hypothesis) is so huge an undertaking for the study of causes of disease that researchers only begin such investigations when they are really necessary to confirm or refute something important. Randomised controlled trials are rarely used for research to detect or to establish causes of disease, mainly because randomisation is most of the time impossible, but quite fortunately, randomisation is most of the time not needed.

## Randomisation: Needed for Intended Effects, Not for Discovery and Explanation

The argument for why randomisation is most of the time not needed in observational research on causes of diseases [9] can be briefly recapitulated by pointing out the contrast between the investigation of beneficial effects versus the investigation of adverse effects of treatments. Beneficial effects are “intended effects” of treatment. In daily medical practice, prescribing will be guided by the prognosis of the patient: the worse the prognosis, the more therapy is given. This leads to intractable “confounding by indication”. Hence, to measure the effect of treatment, we need “concealed randomisation” to break the link between prognosis and prescription [10]. In contrast, adverse effects are “unintended effects” of treatment, and are mostly unexpected and unpredictable, which means that they usually are not associated with the indications for treatment [11]. Thus, there is no possibility of “confounding by indication”, and observational studies on adverse effects can provide data that are as valid as data from randomised trials [12,13]. A straightforward example of an unexpected and unpredictable adverse effect is the development of a rash after prescription of ampicillin in a patient who never used any penicillin derivative or analogue before. The prescribing physician cannot predict this occurrence. Hence, data from routine care in daily practice can be used to study the frequency of such rashes.

This idea can be generalised: most potential causes of disease can be viewed as producing effects that are undesired, unintended, and unexpected [9]. This becomes clear from classic success stories of epidemiologic research: e.g., before the links between smoking and lung cancer or asbestos and mesothelioma were known, people who exposed themselves to these risks were unaware of the consequences—which is why the risks could be investigated by observational studies.

### No blank cheque for observational research.

**Figure 1:**
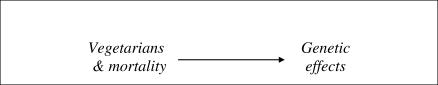
Axis of Haphazardness of Exposure

The above reasoning should not lead to uncritical acceptance of all observational research about causes of diseases. A mental device to guide our judgement about new claims from observational research is to position the research on an “axis of haphazardness of exposure” (Figure 1).

At one side there is research on genetic effects. This is the closest that observational research can come to randomisation. At the other end of the axis there is research contrasting, for example, the mortality of vegetarians to non-vegetarians. That contrast is completely non-haphazard: vegetarians have different social backgrounds, different education, different life styles, and may have taken up the habit because they are health-conscious. The differences in (self) assignment will bias the comparison, and it is known in advance that the bias will be next to intractable in the analysis, since its various components cannot be known in sufficient detail. Therefore, an assessment of the effect of vegetarian diets needs randomised trials, e.g., to show whether vegetarian diets decrease blood pressure.

Most observational research hovers somewhere between the extremes. Sometimes an observational researcher is quite close to the quasirandom haphazardness of genetic exposures, for example, when studying adverse effects in selected groups of patients where the adverse effect is unpredictable [14]. When confronted with a new exposure that is not that close to ideal haphazardness, it is useful to ask oneself whether the most important confounders can be listed, can be measured fairly accurately, and can be controlled for. If the answer to these questions is positive, that will lead to greater credibility of the results. If negative, as in the vegetarian example, we may attach no credibility to the results despite any attempts at statistical correction for confounders.

## Subgroups and Multiplicity of Analyses

Many scientists believe that results from observational research are less credible because of the problem of subgroups and multiplicity of analysis: multiple looks at data for associations that were not the original aims of the data collection.

**Figure 2:**
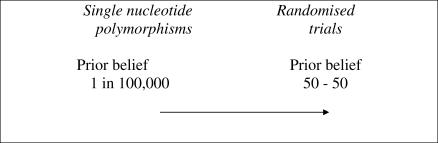
Axis of Multiplicity

This problem can be conceptualised on an “axis of multiplicity” (Figure 2). At one extreme there are genome-wide analyses, where tens of thousands of single nucleotide polymorphisms (SNPs) are investigated for disease associations. The prior probability that some grain of explanation will come from any individual SNP is slim, say, one in 100,000 [15]. At the other extreme, there are randomised trials about a single disease, a single therapy, and a single outcome. Randomised controlled trials are started under equipoise [16,17]: the prior odds that the therapy that is tested is worthwhile are 50–50, and multiplicity of analysis is strictly not allowed. Thus, the axis of multiplicity is at the same time an axis of prior belief: the prior belief that some factor will be a causal explanation for a condition or that some therapy or treatment will work [18].

An often-heard objection about multiplicity in observational research is that many large clinical and epidemiologic data sets exist, and many PhD students analyse these data, which leads to data dredging. However, researchers do not mindlessly grind out one analysis after another [19]. Analyses are guided by clues that involve reasoning, much like in the example of factor V Leiden and oral contraceptives above. That example also shows that we did not “try to explain a subgroup” after we found it. Many people think that researchers find subgroups and then dream up explanations for that finding. The inverse is more likely and more interesting: finding something strange in the data suddenly makes a researcher realise that this could explain some other phenomenon, outside of the data, which was already known but had never been explained before.

In practice, researchers hover over the axis of multiplicity. Sometimes they are closer to the SNPs situation when trying out a bold idea. At other times they are closer to the randomised trial situation with 50–50 prior odds, or they are in an even better a priori position when exploring an association that is well known. For example, a researcher may look at active smoking and lung cancer in data not collected for that purpose. Critics will never say: “You only found that association because of your multiple analyses”. On the contrary, if an association between active smoking and lung cancer were not found, a critic would doubt the validity of the data.

### Hypotheses before or after seeing the data?

Many researchers have the intuition that findings on subgroups that were specified before data analysis are more credible than explanations that arose after seeing the data. In general, the logical proof of this intuition is difficult because new ideas in science often gain credibility when they can explain previous findings that were not understood [20,21].

For randomised trials, this intuition remains useful [22]. Large randomised trials are set up after years of deliberation by dozens of experts. It is not likely that any important prior idea about subgroups in which the therapy might work better or worse was overlooked. Usually this recognition is dealt with by including or excluding such subgroups from the trial. It is therefore unlikely that a new and worthwhile subgroup would turn up during data analysis. Thus, the post hoc discovery of subgroups in randomised trials has low prior probability, from which follows low credibility of subgroup findings.

However, because observational studies concern aetiology, and because aetiologies are often multiple, prior evidence might exist without investigators or data analysts being aware of it. This becomes evident when data are used for new purposes. The Framingham study is an archetypical example: originally started to investigate a few cardiovascular risk factors, it has branched off in many directions, from chronic pulmonary disease to genetics, for which a mix of old and new data are used [23]. When data are used for a different purpose, even if that purpose was found during data analysis, the data acquire new priors, i.e., a different body of literature—even if that literature was not part of setting up the study or the analysis [21,24]. (See example on page 14 in longer version of article in Text S1.)

### Replication: universal solution for multiplicity and subgroup analysis.

Subgroups and multiple analyses are a necessary part of observational research: otherwise, one cannot make new discoveries, nor quickly check discoveries by others. Still, many interesting ideas will have low priors. The universal solution is replication [25]. This was already advocated for subgroups found in randomised trials, where the veracity of a surprising finding can be strongly enhanced if similar subgroup results are found across similar trials in a meta-analysis [22]. In genome-wide analyses, which may have the most severe problems of multiplicity, investigators collaborate in consortia, to replicate findings from genetic analyses as a prerequisite for publication [26].

For observational research, the replication needed is not the simple replication of more or less the same study to obtain larger numbers. When the validity of observational research is doubted, it is usually not because of fear of chance events, but because of potential bias and confounding. Repeating a study in the same way as previous studies may replicate the same problems. Therefore, different studies are needed with different designs, different methods of data collection, and different analyses. This makes systematic reviews of observational studies more difficult, and at the same time more interesting: it is necessary to reason about the advantages and disadvantages of the different studies in the light of potential bias and confounding, and to ponder how one study remedies potential weaknesses of the other [27].

## Rethinking the Hierarchy of Evidence

The ideas about subgroups and prior odds of hypotheses lead to further insight in the usual hierarchy of strength of study designs with the randomised trial on top and the case report at a suspect bottom (Box 1). Perhaps this hierarchy is a hierarchy of prior odds. Intuitively, we may feel that randomised trials are the most robust type of study because positive findings from such trials stand the test of time better than findings from other designs. However, that might be because they start with higher prior odds.

The way in which prior odds might shape our views can be understood when imagining an upside-down world in which randomised trials would be started with the same prior odds of truth as individual SNPs in a genome-wide analysis, say, one in 100,000. Suddenly, randomised trials would look abysmally poor: almost all their positive findings would be chance findings, as one in 20 would be significant by conventional testing. In this upside-down world, almost no positive result of any randomised trial would stand the test of time. Imagine further that observational studies would only be started with priors of at least 50–50. When positive, posterior odds would be of the order of 80–20 or more. Their results would stand the test of time, and would have great face credibility. Observational research would suddenly look very good.

In our real world, randomised trials can solve problems of “confounding by indication” in situations where observational research can not. Still, we may have been deluding ourselves about their unique superiority because they start with much higher prior odds than most observational research. Within the realm of observational research, it is often felt that prospective follow-up studies are stronger than case-control studies. The main argument seems to be that findings of case-control studies are too often not upheld in future studies [6,28,29]. Given that case-control studies will often be the first analytic study of a new idea, while prospective follow-up studies will only be started when something important has to be confirmed—that is, when the prior odds are already higher— this may again explain the difference in seeming strength.

## Synthesis: A Difference in Loss Function?

We need both hierarchies, the hierarchy of discovery and explanation as well as that of evaluation. Without new discoveries leading to potentially better diagnosis, prevention, or therapy, what would we do randomised trials on? Conversely, how could we know that a discovery is useful, if not rigidly evaluated?

The two hierarchies serve different purposes. Many researchers truly enjoy the game of multiplicity of analysis with low priors in observational research: it is the duty of academics to explore hypotheses that are interesting, and to follow them up wherever they lead [30]. The difference with evaluative research might be a difference in “loss function”: the penalty for being wrong. R. A. Fisher once suggested that in contrast to decisions about batches of manufactured products, where one can calculate the penalty for a wrong decision, in science it is impossible to calculate the loss function of a wrongly held or wrongly rejected scientific explanation [31].

Paraphrasing this idea, I propose that the loss function of evaluation research—the prototypical randomised trial of drug therapy—concerns real people who are cured or harmed by our acceptance or rejection of a particular treatment. Under equipoise, the data from randomised trials are the best information that we have. We should not tamper with such data: our delight in exploring new ideas should not be allowed to affect a future patient's health.

In contrast, the loss function of discovery and explanation cannot be defined equally directly. Aetiologic researchers should pursue low-probability hypotheses because these may lead to new insights. Much good can come from going down the wrong alley and detecting why it is wrong, or playing with a seemingly useless hypothesis; the real breakthrough might come from that experience. What is lost if we go too far in the wrong direction is time and money for science. That is again inevitable: science makes progress “in a fitful and meandering way”, as described by Stephen Jay Gould [32].

In the end, we will have to live with the fitful and meandering ways of discovery and explanation, and at the same time call for strict evaluation before we apply new insights to people. There is no other way forward.

## Supporting Information

Text S1 A Longer, More Detailed Version of This Paper (109 KB PDF)

## Abbreviations

SNP: single nucleotide polymorphism

## References

[pmed-0050067-b001] Laine C, Horton R, DeAngelis CD, Drazen JM, Frizelle FA (2007). Clinical trial registration—Looking back and moving ahead. N Engl J Med.

[pmed-0050067-b002] Vandenbroucke JP, Koster T, Briet E, Reitsma PH, Bertina RM (1994). Increased risk of venous thrombosis in oral-contraceptive users who are carriers of factor V Leiden mutation. Lancet.

[pmed-0050067-b003] Vandenbroucke JP, Rosendaal FR, Bertina RM, Khoury JM, Little J, Burke W (2004). Factor V Leiden, oral contraceptives and deep vein thrombosis. Human genome epidemiology.

[pmed-0050067-b004] Vandenbroucke JP, Rosing J, Bloemenkamp KW, Middeldorp S, Helmerhorst FM (2001). Oral contraceptives and the risk of venous thrombosis. N Engl J Med.

[pmed-0050067-b005] Vandenbroucke JP, Kroep JR (2005). Bortezomib in multiple myeloma. N Engl J Med.

[pmed-0050067-b006] Tugwell P, Haynes RB, Sackett DL (1985). Clinical epidemiology: A basic science for clinical medicine.

[pmed-0050067-b007] Glasziou P, Chalmers I, Rawlins M, McCulloch P (2007). When are randomised trials unnecessary? Picking signal from noise. BMJ.

[pmed-0050067-b008] Vandenbroucke JP (2001). In defense of case reports and case series. Ann Intern Med.

[pmed-0050067-b009] Vandenbroucke JP (2004). When are observational studies as credible as randomised trials. Lancet.

[pmed-0050067-b010] Schulz KF, Grimes DA (2002). Allocation concealment in randomised trials: Defending against deciphering. Lancet.

[pmed-0050067-b011] Miettinen OS (1983). The need for randomization in the study of intended effects. Stat Med.

[pmed-0050067-b012] Vandenbroucke JP (2006). What is the best evidence for determining harms of medical treatment. CMAJ.

[pmed-0050067-b013] Papanikolaou PN, Christidi GD, Ioannidis JP (2006). Comparison of evidence on harms of medical interventions in randomized and nonrandomized studies. CMAJ.

[pmed-0050067-b014] Jick H, Vessey MP (1978). Case-control studies in the evaluation of drug-induced illness. Am J Epidemiol.

[pmed-0050067-b015] Wellcome Trust Case Control Consortium (2007). Genome-wide association study of 14,000 cases of seven common diseases and 3,000 shared controls. Nature.

[pmed-0050067-b016] Djulbegovic B, Lacevic M, Cantor A, Fields KK, Bennett CL (2000). The uncertainty principle and industry-sponsored research. Lancet.

[pmed-0050067-b017] Kumar A, Soares H, Wells R, Clarke M, Hozo I (2005). Children's Oncology Group. Are experimental treatments for cancer in children superior to established treatments? Observational study of randomised controlled trials by the Children's Oncology Group. BMJ.

[pmed-0050067-b018] Rothman KJ (1990). No adjustments are needed for multiple comparisons. Epidemiology.

[pmed-0050067-b019] Greenland S (2007). Commentary: on “quality in epidemiological research”: Should we be submitting papers before we have the results and submitting more “hypothesis generating research. Int J Epidemiol.

[pmed-0050067-b020] Lipton P (2005). Testing hypotheses: prediction and prejudice. Science.

[pmed-0050067-b021] Brush SG (2005). Accommodation or prediction. Science.

[pmed-0050067-b022] Rothwell PM (2005). Treating individuals 2. Subgroup analysis in randomised controlled trials: Importance, indications, and interpretation. Lancet.

[pmed-0050067-b023] Joost O, Wilk JB, Cupples LA, Harmon M, Shearman AM (2002). Genetic loci influencing lung function: A genome-wide scan in the Framingham Study. Am J Respir Crit Care Med.

[pmed-0050067-b024] Goodman SN (1998). Multiple comparisons, explained. Am J Epidemiol.

[pmed-0050067-b025] Moonesinghe R, Khoury MJ, Janssens ACJW (2007). Most published research findings are false—But a little replication goes a long way. PLoS Med.

[pmed-0050067-b026] Khoury MJ, Little J, Gwinn M, Ioannidis JP (2007). On the synthesis and interpretation of consistent but weak gene-disease associations in the era of genome-wide association studies. Int J Epidemiol.

[pmed-0050067-b027] Maclure M (1993). Demonstration of deductive meta-analysis: Ethanol intake and risk of myocardial infarction. Epidemiol Rev.

[pmed-0050067-b028] Mayes LC, Horwitz RI, Feinstein AR (1988). A collection of 56 topics with contradictory results in case-control research. Int J Epidemiol.

[pmed-0050067-b029] Schulz KF, Grimes DA (2002). Case-control studies: Research in reverse. Lancet.

[pmed-0050067-b030] Ignatieff M (2007 August 5). Getting Iraq wrong. The New York Times Magazine.

[pmed-0050067-b031] Fisher RA (1973). Statistical methods and scientific inference.

[pmed-0050067-b032] Gould SJ (2000). Pathways of discovery: Deconstructing the “science wars” by reconstructing an old mold. Science.

