# Supplementary material for: Observational Research, Randomised Trials, and Two Views of Medical Science
Source: PLoS Med. 2008 Mar 11;5(3):e67. doi: 10.1371/journal.pmed.0050067 (PMC2265762; doi:10.1371/journal.pmed.0050067)
Supplement: Supplementary file 1 [file pmed.0050067.sd001.pdf]

Supporting information file S1: Longer, more detailed version of article

Observational research, randomised trials and two views of medical science *

Jan P Vandenbroucke, MD, PhD, FRCP, FRCPE

Professor of Clinical Epidemiology and Academy Professor of the Royal Netherlands Academy of Arts and Sciences

Department of Clinical Epidemiology
Leiden University Medical Centre, 1- C9-P
PO Box 9600, 2300 RC Leiden, The Netherlands
Phone: +31-71-5265230, Fax: +31-71-5266994
Email: j.p.vandenbroucke@lumc.nl

Draft: 19 February, 2008

***This document is a more complete version of the Austin Bradford Hill Memorial Lecture, given at the London School of Hygiene and Tropical Medicine on 24 April 2007. The lecture text was amended because of comments by discussants and three reviewers of PLoS Medicine. The first seven pages of this longer version are close to the main paper in PLoS Medicine. Thereafter, the longer text treats more topics and gives more examples.**

Two views of medical science

Two views about medical research seem to have split ever more apart over the past decades. One view is that of medical researchers who rejoice in discoveries and explanations of causes of disease. Discoveries happen when things are suddenly seen in another light. Ideas strike by seeing the odd course of a disease in a patient, the strange results of a lab experiment, a peculiar subgroup in the analysis of data, or some juxtaposition of papers in the literature. Researchers continuously have masses of ideas. They get enthusiastic about one idea, and will try to find data to see whether there is "something in it". For first exploration, they will preferably use existing clinical or epidemiologic data, do a quick additional lab experiment, a quick search for more literature, or look for some more patients. As soon as there is a hint of confirmation, a paper is submitted. The next wave of researchers reads this paper and immediately tries to check this idea, using their own existing data or their trusted lab experiments. They will grill the idea by looking at different subgroups of diseased persons, by varying the definition of exposures, by taking potential bias and confounding into account, or by varying the lab conditions to explain why the new idea holds – or why it is patently wrong. In turn they swiftly submit their results for publication. These early exchanges may lead to strong confirmation or strong negation. If not, new studies are needed to bring a controversy to resolution.

The other view is that of medical researchers whose aim is to set up studies to evaluate whether the patient's lot is really improved by the new therapies, diagnostics and insights that looked so wonderful in the lab or on initial testing? The most developed branch of evaluation research is randomised trials of drug therapy, which I will use as its prototype in this paper. One major condition for credibility of such trials is complete preplanning of every aspect of the trial, and nowadays even advance registration and documentation of everything that was preplanned [1]. This preplanning should not be strayed from, however promising some side alley looks, because the credibility of the results will immediately take a nose dive.

What they think about each other

From the perspective of the evaluative researcher, this method of discovery and explanation is dangerously biased: clinicians present case series out of the blue, epidemiologists do multiple analyses on existing data collected for completely different purposes, basic scientists repeat

lab experiments with endless new variations, changing the hypothesis as well as the experiment continuously, until something fits. And all these researchers always dream up perfect explanations. This leads to irresponsible “hypes” and “scares” in the popular press, and to unnecessary research loops that are a burden to the pubic purse.

In contrast, the discovery type of researcher is convinced that evaluation is not just hopelessly dull, but that too much emphasis on evaluation actually hampers the progress of science - precisely because everything is preplanned. For discovery you need chance and one-sided views. You need to look at the literature in a slanted way, to examine data of others as well as your own to see them in a different light. To discoverers, evaluation is mainly a form of “quality control” that society needs for financial reimbursement by third party payers, but it is not truly science. And finally: numbers are not explanations. That idea was already expressed by Trousseau, in France in the 1850s [2], in his polemic opposition to numerical medicine. Numbers do not tell a story of what produces what and why it does so. Numbers do not give insight upon which you can build the next step of your reasoning, i.e., your next investigation, your next application in patients, or to understand what is happening in a particular patient. Still today, this is the feeling of the discovery-type scientist towards numerical evaluation.

Co-existence in the mind of an individual?

Yet, these two views of medical research can exist simultaneously in the mind of one person. Over the past decades, I may have made one contribution to unravel the aetiology of a disease: the detection of the interaction between factor V Leiden and oral contraceptives in causing venous thrombosis [3]. Young women who carry the factor V Leiden mutation (about 5% of the population of white European descent) and also use oral contraceptives have a much higher risk of venous thrombosis than women with either risk factor alone (Table 1).

Table 1 - Analysis of oral contraceptive use, presence of factor V Leiden allele, and risk for venous thromboembolism.

Factor V Leiden	Oral Contraceptives	Number of Patients	Number of Controls	Odds Ratio (rounded)
Yes	Yes	25	2	35
Yes	No	10	4	7
No	Yes	84	63	4
No	No	36	100	1 (Reference group)

*Modified from Vandenbroucke et al. [3]

This finding was not at all preplanned. Our study originally aimed at quantifying existing biochemical and genetic risk factors for venous thrombosis. The factor V Leiden mutation, a new risk factor for venous thrombosis, was discovered during the study by biochemical means, in part through data from the study. After the mutation was established, we looked again at the data which included patients and controls of both sexes from age 15 to 70, and found a few homozygotes for the Factor V Leiden mutation among the patients. To our surprise, almost all these homozygotes with venous thrombosis were young women who used oral contraceptives [4]. This was our moment of discovery. Since the early 1960s it was known that some women develop venous thrombosis when using oral contraceptives, but no mechanism ever stood out as a possible explanation. We felt that our data might be the beginning of an understanding and we analysed homozygotes and heterozytoges together for the interaction with factor V Leiden (Table 1). The findings provided insight into the question of why exogenous hormones cause venous thrombosis, and were the source of much subsequent research [5].

However, whenever I suspect that a report from a randomised controlled trial has strayed from the path of complete preplanning, e.g. by having highlighted some subgroup in the analysis or by cutting corners in the completeness of the follow-up, I might be the first to cry “beware” [6]. While the two views on medical research lead to completely different mindsets about subgroups and exploring new findings in data, I do teach and encourage both to young researchers.

Different hierarchies for different problems

Underlying the difference in views are differences in the hierarchy of research designs that apply to different research problems.

A hierarchy of "strength" of research designs with the randomised trial on top and the anecdotal case report at a suspect bottom is well known. It has been used since the 1980s in various guises [7] and under various names, but a rather typical rendering is shown in Box 1. I have qualified this hierarchy by naming it the hierarchy of study designs for *"intended effects of therapy"*, i.e., the beneficial effects of treatments that are hoped for at the start of a study.

Box 1: Hierarchy of study designs for *intended effects of therapy*

1.Randomised controlled trials
2.Prospective follow-up studies
3.Retrospective follow-up studies
4.Case-control studies
5.Anecdotal: case reports and series

The opposing hierarchy ranks study designs in the order in which they give the best chances of discovery and of studying new explanations, and is shown in Box 2.

Box 2: Hierarchy of study designs for *discovery and explanation*

1.Anecdotal: case reports and series, findings in data, literature
2.Case-control studies
3.Retrospective follow-up studies
4.Prospective follow-up studies
5.Randomised controlled trials

The entries in the second hierarchy are almost the same, except that the ranking is reversed. The first entry is somewhat enlarged, as anecdotal reports that lead to new ideas comprise not only case descriptions of patients, but also discoveries in data and juxtaposition of ideas in the

literature. Any clinician or laboratory researcher will immediately recognise that this is the sequence of how new discoveries are made: the odd course of disease in a patient, a remarkable lab result, a peculiar subgroup such as the factor V Leiden homozygotes, or a finding in a seemingly unrelated part of the literature, spark a new idea. Only thereafter do analytic research designs come into play. Some examples: genetic research begins with an interesting family tree, infectious disease outbreak investigations begin with a few cases that come to the attention of a doctor and provide the first clues of transmission, the first signal of adverse effects of therapy is more often than not from individual observations by astute patients or physicians [8], as are the first ideas about the harms of occupational exposures [9].

A juxtaposition of the hierarchies

In both hierarchies, there are large gaps of credibility and usefulness between the different levels. For evaluation of the intended effects of therapy, the randomised controlled trial stands out, followed at quite a distance by all observational designs. The second in line, the prospective follow-up is already suspect for the evaluation of therapy because any observational study of intended outcomes has nearly intractable problems of confounding by indication. Only very rarely we will believe case reports or series as evidence for therapy, for instance when effects are dramatic, which means that we have a secure feeling for a “mental control group”, and the deviation from the mental control group is large [10, 11].

For discoveries, the original case reports, lab observations, data analysis, or juxtaposition in the literature may be so convincing that they stand by themselves, either because of the magnitude of the effect or because the new explanation suddenly and convincingly makes the new finding fall into place with previous unexplained data or previous ideas. In most instances, however, we need analytic studies to see whether the observation really holds. The preferred designs of researchers are case-control studies, or possibly retrospective follow-up studies (where the exposure and follow-up experience lies in the past from the point of view of the researcher when she had her new idea), because such designs will give the quickest answer for the least effort. If at all possible, researchers will use existing data. For many problems in genetics, for infectious disease outbreaks, or for adverse effects of drugs no further evidence may be needed. A truly prospective follow-up study (i.e., involving new data collection and start of follow-up after the formulation of the specific hypothesis) is so huge an undertaking for the study of causes of disease - as most diseases are relatively rare - that

researchers only begin such investigations when they are really necessary to confirm something important. Even for prognostic studies into the course of disease in patients, retrospective follow-up studies or case-controls studies on existing data are often preferred – although a prospective study might be more feasible when the disease endpoints among the patients is sufficiently frequent. Randomised controlled trials are rarely used for research to detect or to establish causes of disease. Randomised trials are by definition set up as verification, and not to detect new causes of disease: any discovery in a randomised trial is accidental and might need a new investigation. Most importantly, randomisation is usually impossible to study causes of disease, but quite fortunately, randomisation is most of the time not needed.

Randomisation: needed for intended effects, not for discovery and explanation

I have presented previously the argument for why randomisation is most of the time not needed in observational etiologic research of causes of diseases [12]. This can be briefly recapitulated by pointing out the contrast between the investigation of beneficial effects versus the investigation of adverse effects of treatments. Beneficial effects are “intended effects” of treatment. In daily medical practice prescribing will be guided by the prognosis of the patient: the worse the prognosis the more therapy is given. This leads to “confounding by indication” that is intractable. Hence, to measure the effect of treatment, we need “concealed randomisation” to break the link between prognosis and prescription [13]. Concealed randomisation guarantees that the act of allocating treatments is unbiased for prognosis – it does not guarantee equality of prognostic factors but instead guarantees that any difference arises by chance.

In contrast, adverse effects are “unintended effects” of treatment, and are mostly unexpected and unpredictable. Therefore, adverse effects are usually not associated with the indications for treatment [14]. In such circumstances, there is no possibility of “confounding by indication”, and observational studies on adverse effects can provide data that are as valid as data from randomised trials. Thus, data from daily practice can be used for research. A straightforward example of an unexpected and unpredictable adverse effect is the development of a rash after prescription of ampicillin in a patient who never used any penicillin derivative or analogue before. The prescribing physician cannot predict this

occurrence, and a study with data from daily practice suffices to investigate the frequency of such rashes.

To rule out any residual “confounding by indication” as much as possible, observational studies can be refined, e.g. by limiting studies to *idiopathic* cases of the disease that is the potential adverse effects of a drug: patients who have no risk factor for the adverse effect that could in any way have guided treatment [15]. For example, a study on the risk of venous thrombosis with different types of oral contraceptives can be restricted to young women without any risk factor for venous thrombosis. It is reasonable to assume that, because the choice of oral contraceptive could not be guided by known risk factors - as there were no risk factors at the time of prescribing - any difference in rates of venous thrombosis can be ascribed to a difference in the contraceptive [15, 16]. In the absence of risk factors, the choice for different types of pill could have been guided by anything, such as convenience, cost, practice guidelines, or the latest visit by an industry representative, which are not risk factors for venous thrombosis. The “pseudo-randomness” of the allocation to diverse types of pills, given this selection of idiopathic cases, only applies to the outcome of venous thrombosis. It may not apply to other outcomes. In general, the assumption works best when the disease that is the adverse effect is a totally different disease from the one that is treated [12]. Of course, it may still be necessary to adjust for potential confounders that may arise, such as a difference in age between users of different types of oral contraceptives [12]. In a similar way, in randomised trials adjustment for baseline imbalances may be necessary when such imbalances occur by chance.

An empirical evaluation of the idea that adverse effects can be investigated validly by observational studies was given by a comparison of the findings on the same adverse effects between large meta-analyses of randomised trials and large observational studies [17]. This comparison found no overall predilection for either design to lead to higher or lower effect measure estimates. If anything, observational studies were more conservative: on average they yielded somewhat lower excess risks of harm than randomised trials. In the few instances where observational studies yielded much larger relative risks than randomised trials, the observational data were likely to reflect actual prescribing to a less selected group of patients than had been enrolled in the trials - which made the observational data a better reflection of the real harms suffered by patients [16, 17]. This comparative study was only possible for a limited number of adverse effects, because the adverse effect needed to be relatively frequent,

must have occurred rather soon after initiation of treatment, and must have been thought about beforehand in the randomised trials. Despite these limitations, the comparison is compatible with the idea that observational studies on adverse effects can be equally credible as randomised studies.

Generalisation to observational studies on causes of disease

Almost all potential causes of disease can be viewed as producing effects that are undesired, unintended, and unexpected [12]. This becomes clear from classic success stories of epidemiologic research: e.g., before the links between smoking and lung cancer or asbestos and mesothelioma were known, people who exposed themselves to these risks were unaware of the consequences– which is why the risks could be investigated by observational studies. Some researchers hold the view that these effects stood out because they are large. However, other epidemiologic classics concerned much lower relative increases in risk: e.g., lead in indoor paint and the mental development of children, or age at first pregnancy and the development of breast cancer.

No blank cheque for observational research

The above reasoning should not lead to uncritical acceptance of all observational research about causes of diseases. A mental device to guide our judgement about new claims from observational research is to position the research on an “axis of haphazardness of exposure” (Figure 1).

Figure 1: Axis of haphazardness of exposure

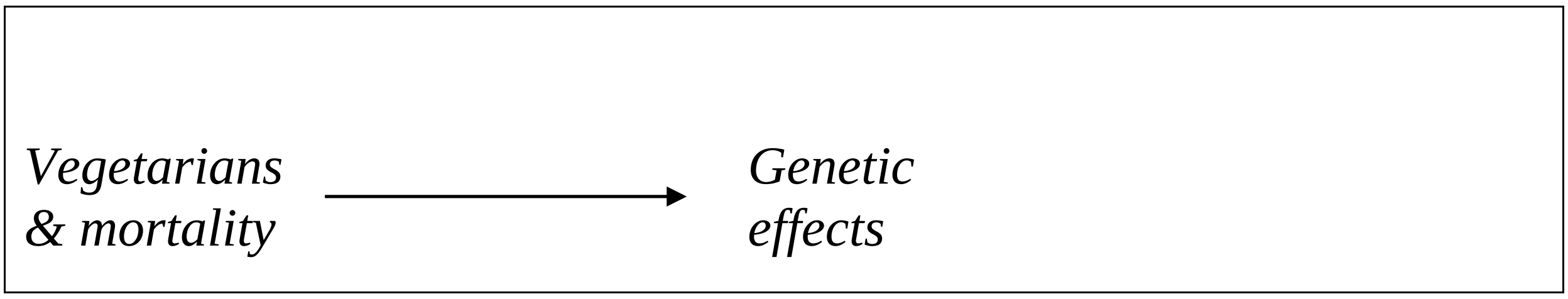

At one side there is research on genetic effects. Most researchers accept that this is the closest that observational research can come to randomisation – even if “Mendelian randomisation”, i.e. the independent assortment of genes on different chromosomes when gametes are formed, is a biological mechanism and therefore not exactly the same as physical randomisation [18]. At the other end of the axis there is research contrasting, for example, the mortality of vegetarians to non-vegetarians. That contrast is completely non-haphazard: vegetarians have different social backgrounds, different education, different life styles, and may have taken up the habit because they are health-conscious which makes the health effects “intended”. The differences in (self) assignment of the vegetarian diet will bias the comparison and it is known in advance that the bias will be next to intractable in the analysis, since the various components of this bias cannot be known in sufficient detail. Therefore, an assessment of the effect of vegetarian diets needs randomised trials, e.g., to show whether vegetarian diets nibble off some millimetres of mercury from blood pressure. If an effect on blood pressure is found, this might be a good reason to advocate a more vegetarian diet. Observational studies on mortality, however, cannot be the basis for such recommendations because of their low credibility. Even if the possibility exists that part of a reduced mortality is due to blood pressure reduction, that effect cannot be credibly separated from all confounding effects in an observational study.

Most observational research hovers somewhere between the extremes. Sometimes an observational researcher is quite close to the quasi-random haphazardness of genetic exposures, for example, when studying adverse effects in selected groups of patients where the adverse effect is unpredictable. When confronted with a new exposure that is not that close to ideal haphazardness, it is useful to ask oneself whether the most important confounders can be listed, can be measured fairly accurately, and can be controlled for. If the answer to these questions is positive, that will lead to greater credibility of the results. If negative, as in the vegetarian example, we may attach no credibility to the results despite any attempts at statistical correction for confounders.

Unexpected beneficial effects?

A difficult area is the unexpected beneficial effects of medical treatments, e.g. beneficial effects of drugs on organ systems, other than their original targets, that were not foreseen. A priori, they are less likely, as it is not likely that a new external agent will suddenly improve

the human constitution that has evolved over tens of thousands of years; external agents are more likely to wreck havoc in this finely tuned biologic machinery [12]. Still, an unexpected beneficial effect is possible, as was shown by the discovery of the preventive effect of aspirin on heart disease [19]. However, the study of unexpected beneficial effects, seems to suffer more often from problems of selection than adverse effect research. Amongst others, a "healthy user bias" may exist. For example, among cardiovascular patients, those who use their statins diligently are a positive selection, for cardiovascular risk as well as in many other respects [20-22]. In observational studies statins have been described to protect from dementia as well as from fractures, which was not confirmed in randomised trials [16]. Likewise, an apparent protection from pneumonia by statins was explained because statin users also received pneumococcal vaccinations more often [23]. The health intentions that accompany statin use wreck the possibility to study beneficial effects. In contrast, when major diseases are an adverse effect of therapy, such as myocardial infarction with Cox-2 inhibitors, observational evidence gave the same results as randomised [24].

In sum, observational research on humans is most credible when it concerns negative unexpected effects. Sufficient haphazardness of exposure allocation offers an admittedly subjective guide to the credibility of observational research findings. However, in the words of Rosenbaum: *"Haphazard is not random.... Still, haphazard or ostensibly irrelevant assignments are to be preferred to assignments which are known to be biased in ways that cannot be measured and removed analytically."* [25, page 345]

Subgroups and multiplicity of analyses

Even if it is accepted that physical randomisation may not be necessary, many scientist still feel that results from observational research are less credible because of the problem of subgroups and multiplicity of analysis: multiple looks at data for associations that were not the original aims of the data collection.

This problem can be conceptualised on an "axis of multiplicity" (Figure 2).

Figure 2: Axis of multiplicity

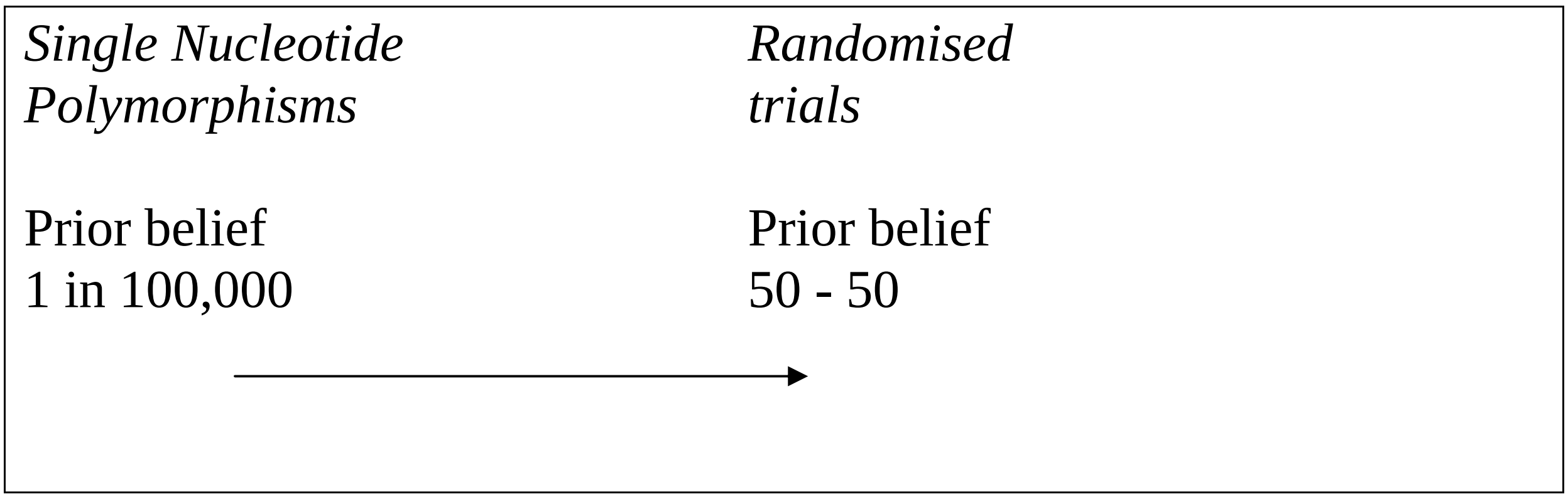

At one extreme there are genome-wide analyses, where tens of thousands of single nucleotide polymorphisms (SNPs) are investigated for disease associations. The prior probability that some grain of explanation will come from any individual SNP is slim, say, 1 in 100,000 [26]. At the other extreme, there are randomised trials about a single disease, a single therapy, and a single outcome. Randomised controlled trials are started under equipoise [27, 28]: the prior odds that the therapy that is tested is worthwhile are 50-50, and multiplicity of analysis is strictly not allowed. Thus, the axis of multiplicity is at the same time an axis of prior belief: the prior belief that some factor will be a causal explanation for a condition or that some therapy or treatment will work [29]. In general, when many exposures are investigated, most will have low priors; conversely, when only a singly exposure and outcome is studied, the study is often started with a much higher prior.

An often-heard objection about multiplicity in observational research is that many large clinical and epidemiologic data sets exist, and many PhD students analyse these data, which leads to data dredging and hunting for significance. However, “many PhD students looking at data” is not the same situation as the analysis of tens of thousands of SNPs. PhD students do not mindlessly grind out one analysis after another [30]. A PhD student often starts by verifying tentative ideas from previous publications; next she will look whether she can form new insights herself. The clues that guide the analysis involve reasoning, much like in the example of factor V Leiden and oral contraceptives above. That example also shows that we did not “try to explain a subgroup” after we found it. Many people think that researchers find

subgroup and then dream up explanations for that finding. While this happens, the inverse is more likely and more interesting: finding something strange in the data suddenly makes a researcher realise that this could explain another phenomenon, outside of the data, which was already known but had never been explained before. Thereby, the explanation reaches out to the existing literature.

In practice, PhD students hover over the axis of multiplicity. Sometimes they are closer to SNPs when trying out a bold idea. At other times they are closer to the randomised trial situation with 50-50 prior odds, or they are in an even better a priori position when exploring an association that is well known. For example, a PhD student may look at active smoking and lung cancer in data not collected for that purpose. Critics will never say: “You only found that association because of multiple analyses by many PhD students”. On the contrary, if an association between active smoking and lung cancer were not found, a critic would doubt the validity of the data – rightfully - so strong is the prior belief.

Hypotheses before or after seeing the data?

Many researchers have the intuition that findings on subgroups that were specified before data analysis are more credible than explanations that arose after seeing the data. In general, the logical proof of this intuition is difficult [31]. The difference between the confirmation of an hypothesis that was specified beforehand and the confirmation of ideas that are constructed during data analysis is much smaller than is usually believed, because new explanations in science often gain most of their credibility when they can explain previous findings that were not understood [32].

Again, the ways of observational and randomised research may split. For randomised trials, this intuition remains useful [33]**.** Large randomised trials are set up after years of deliberation by dozens of experts. As the trial concerns one therapy, one disease and one outcome, it is not likely that any important prior idea about subgroups in which the therapy might work better or worse was overlooked in the planning stage. Usually this recognition is dealt with by including or excluding such subgroups from the trial. It is therefore unlikely that a new and worthwhile subgroup would turn up during data analysis. Thus, the post hoc discovery of subgroups in randomised trials has low prior probability, from which follows low credibility of subgroup findings.

However, because observational studies concern aetiology, and because aetiologies are often multiple, prior evidence might exist without investigators or data-analysts being aware of it. This becomes evident when data are used for new purposes. The Framingham study is an archetypical example: originally started to investigate a few cardiovascular risk factors, it has branched off in many directions, from chronic pulmonary disease to genetics, for which a mix of old and new data are used [34].

When data are used for a different purpose, even if that purpose was found during the data analysis, the data acquire new priors, i.e., a different body of literature - even if that literature was not part of setting up the study of the analysis [32, 35]. Such a change in prior information happened to me when investigating a case series of autopsies of patients who had died of idiopathic pulmonary emboli. The original aim of the study was to determine the frequency of factor V Leiden in the DNA of the autopsy material of a series of deceased patients. Unexpectedly, when looking at the autopsy summaries, about one third were found to be psychiatric patients, and most were apparently medically treated [36]. When turning to the literature it was found that in the early days of neuroleptic drug use in German psychiatric clinics, there had been multiple reports about an increase in pulmonary emboli following the introduction of these drugs – a body of literature that was forgotten [37]. At the time of our investigation (around 1997), a new study was published with an unexpected and unexplained large relative risk for pulmonary emboli for a new antipsychotic agent [38]. Thus, the finding in our case series acquired prior knowledge from the older literature and from the newer study. Taken together, this was reasonable evidence and the line of investigation was continued by others in existing pharmacoepidemiologic databases [39]. Again, this example shows that the unexpected finding led to an idea that enabled the incorporation of existing knowledge and thereby gained credibility.

Replication: universal solution for multiplicity and subgroup analysis

Subgroups and multiple analyses are a necessary part of observational research: otherwise, one cannot make new discoveries, nor quickly check discoveries by others. Still, many interesting ideas will have low priors. The universal solution is replication [40]. Systematic reviews and meta-analysis may play a major role in formalising replication. This was already advocated for subgroups in randomised trials, which have low priors and are usually

presented with severe caution, emphasising the post hoc nature of the finding. The veracity of a surprising finding in a post-hoc randomised trial subgroup can be strongly enhanced if similar subgroup results are found across similar trials in a meta-analysis [33]. In genome-wide analyses, which may have the most severe problems of multiplicity, investigators often collaborate in consortia, to replicate findings from genetic analyses as a prerequisite for publication [41].

In observational research, an original report on a new finding should give a candid account about the circumstances in which the finding arose. For example, it may be important that readers know what the aim was of the original study, because this tells which variables were the original focus, and might therefore have been measured best [42]. If the new finding arises from a re-analysis of existing data, the reader might be informed what prespecified question led to the re-analysis, even if during that analysis something else was found. The authors might indicate what prior evidence existed (even if found after the analysis), and reflect self-critically upon the validity and reliability of the measurements that were not the primary focus of the data collection. This helps to inform the reader where on the axis of multiplicity - the axis of prior belief - the authors have been operating.

For observational research, it is important to realise that the replication that is needed is not a "simple replication" of the same type of study to obtain larger numbers. When the validity of observational research is doubted, it is usually not in the first place because of fear of chance events, but because of potential bias and confounding. Repeating a study in more or less the same way as previous studies may replicate the same problems. Therefore, different studies are needed with different designs, different methods of data collection, and with different analyses, to tackle potential problems of previous studies. This makes systematic reviews of observational studies more difficult, and at the same time more interesting: it does not suffice to amass research to obtain larger numbers, but it is necessary to reason about the advantages and disadvantages of the different studies, and to ponder how one study remedies potential weaknesses of the other [43].

What is needed to convince an audience of a new finding, may also depend on the "sensitivity" (cultural or other) of the finding: if a new finding challenges established beliefs, more and stronger evidence may be needed than if the new finding fits very well with current beliefs, which may lead to rapid acceptance.

Publication bias: registration for observational research?

When only positive findings come to print, multiplicity of analysis may lead to "publication bias". For randomised trials this has been addressed by mandatory registration which permits to track the record of all trials that were started as well as their originally intended endpoints and analyses [1]. Could the same work for observational research? The difference becomes obvious when thinking about studies whose aims change or when additional analyses are done on existing data because of new ideas. It is impossible to register every fleeting thought in the head of the person analysing data, in particular when the thought is rejected at the first pass. Thus, replication remains the only possibility for observational research.

A different issue would be to register which variables are available in major epidemiologic and clinical studies – whether or not the variables (exposures and outcomes) were the primary aim of the study – so that others might benefit from that knowledge, to try out a new idea, to attempt replications, or to wonder why a particular analysis was never done or never published [30]. The "cohort profiles" published by the *International Journal of Epidemiology* come some way in this direction [44].

Rethinking the hierarchy of evidence

The ideas about subgroups and prior odds of hypotheses lead to further insight in the background for the usual hierarchy of strength of study designs – with the randomised trial on top and the case report at a suspect bottom (Box 1). This hierarchy may actually be a hierarchy of prior odds. Intuitively, we may feel that randomised trials are the most robust type of study because positive findings from such trials stand the test of time better than findings from other designs. We think that this is because of their superior design, but perhaps they are more robust because they start with higher prior odds.

The importance of the prior odds at which research is started was highlighted in a paper describing "Why most research findings are false" [45]. In the calculations in that paper, randomised trials start with high prior odds of truth: 50-50 which is an ethical necessity under equipoise [27, 28]. In contrast, all observational studies are given much lower prior odds (1:10

or less, especially the discovery oriented studies). Because most of the literature is observational, most priors in the literature will be way below 50-50. In itself that makes it more difficult to achieve a more than 50% posterior probability of truth. Also, in the paper, a generous dose of potential bias and confounding is added to observational research. In a certain sense the reasoning is circular because the combination of low prior odds with bias and confounding will always lead to the conclusion that a majority of so-called positive research findings are false [46]. However, it does suggest why much research concerning new discoveries or new explanations - which will inevitably have low prior odds - will not be upheld by future research.

The way in which prior odds might shape our views about the strength of research designs can be understood when imagining an upside-down world in which randomised trials would be started with the same prior odds of truth as individual SNPs in a genome-wide analysis, say, 1 in 100,000. Suddenly, randomised trials would look abysmally poor: almost all their positive findings would be chance findings, as 1 in 20 would be significant by conventional testing. In this upside-down world, almost no positive result of any randomised trial would stand the test of time. Imagine further that observational studies would only be started with priors of at least 50-50. When positive, posterior odds would be of the order of 80-20 or more. Their results would stand the test of time, and would have great face credibility. Observational research would suddenly look very good. In such a world, we would readily find explanations for this difference in credibility: we might feel that the allocation to the groups in randomised trials was only a game of chance, while in observational studies much thinking went into defining which groups to contrast, and we might feel that this made these studies superior.

The above reasoning should not let us lose sight of the idea that randomisation can indeed solve the problem of confounding by indication in circumstances where observational studies can not. Still, this particular advantage does not lead to universal superiority for all types of research questions in all circumstances. This idea was stated already in 1954 by Jerome Cornfield: *"There are no such categories as first-class or second-class evidence. There are merely associations, whether observational or experimental that, in a given state of knowledge, can be accounted for in only one way or in several different ways."* [47]

The role of case-control studies

Within the realm of observational research, there exists a near universal idea that prospective follow-up studies give stronger evidence than retrospective ones and they in turn stronger than case-control studies. A truly prospective follow-up into causes of disease (with new baseline data collection and follow-up after a hypothesis is formulated), however, is only started when there is strong prior opinion that something important will be found, or when it is deemed necessary to convince others.

Sir Richard Doll described how he was invited in 1947 by Sir Austin Bradford Hill for the first formal study on smoking and lung cancer in the UK, which was a case control study [48]. It was set up and analysed admirably – even with a shrewd method of approximating the relative risk without using the odds ratio – as the odds ratio had not yet been invented [49]. Later, Austin Bradford Hill proposed a follow-up study in doctors, not to convince himself or other researchers in the field, but to convince others – the follow-up study was started with a high prior [48]

The case-control study is often the first analytic study after an original idea has been formulated. Thereby the case-control study is the universal work-horse of observational research, the “default” of many an etiologic researcher [50]. Researchers constantly have masses of ideas. Case-control studies are the most expedient form of research in terms of time and money. They can be set up as completely new studies or nested in existing follow-up data, like in pharmacoepidemiologic databases that link medication use with outcomes. Case-control studies yield the same rate ratio estimate as a follow-up study if care is taken about the choice of the control group [51]. Case-control studies often allow better assessment of outcome, as each diseased person can be investigated soon after diagnosis which is often impossible in large follow-up studies (where disease outcomes are reported by participants and investigators have to rely on diagnoses from other hospitals or from a registry). Case-control studies also often permit better assessment of exposure, because they allow the application of more focused time and money. For recent exposures, e.g. in the months before disease develops, they are the only possibility. Thus, case-control studies have at least the same, and in some circumstances greater validity than follow-up studies. They often suffice in

themselves for many subjects in pathogenesis and aetiology, e.g., for genetics, for outbreak investigations, for many environmental and occupational exposures and for studies of adverse effects of drugs. The few instances in which they cannot be applied are when some etiologic factor changes after disease onset, or when the evolution of some parameter has to be followed over time. Despite all their advantages, case-control studies have a bad press. It is often believed that they have a greater potential for bias. The basic argument seems to be that their findings are too often not replicated [7, 52, 53]. That is inevitable, however, when they are the first analytic study of a new idea.

Large vs. small relative risks

Some scientists believe that the results of observational research are only really trustworthy if relative risks are large, e.g. larger than 3, and that smaller relative risks should be eyed with suspicion. This notion is based on an epochal paper by Cornfield et al. about the 1950s smoking and lung cancer controversies [54]. The paper proposed that it is difficult to think of potential confounders to explain a 9-fold relative risk of smoking on lung cancer incidence because potential confounders should be even more strongly associated with smoking. That does not mean that such confounders cannot exist, but that it is difficult to come up with likely candidates to explain away such a large relative risk. However, the inverse is not true: it is not because a relative risk is small that it is untrustworthy. More candidate confounders can be imagined, but that does not mean that the association is in fact confounded. Large relative risks may stand by themselves, and may need little additional evidence. Smaller relative risks may need more epidemiologic evidence, from repeated studies trying to tackle potential bias and confounding. In accepting small relative risks as credible, the idea that the allocation of the exposure could not really be confounded, i.e., that the exposure was sufficiently haphazard, may also play a role. Small relative risks often need additional evidence from other lines of research, e.g., experimental evidence from basic science about mechanisms.

Several recent adverse effects findings that strongly influenced drug prescribing involved relative risks that were small, e.g., about Cox-2 inhibitors and myocardial infarction, and the difference in thrombosis risk between second and third generation oral contraceptives which was upheld by coagulation evidence [5]. Relative risks from SNPs found in genome wide analyses are also usually quite small – yet, are held to be credible if replicated, and in particular if they point to genes that are likely candidates to have a role in the disease that is

studied, which is a “prior” that is established after finding a SNP that stands out statistically [41].

In thinking about the role of basic science in supporting epidemiologic findings, it is often forgotten that the relation between evidence from basic science and from numerical data analysis is a two-way process. Epidemiologic findings can become acceptable because of basic science findings that explain mechanisms, but the inverse is also true: a basic science finding would have no meaning – i.e., would never be an explanation - if we did not know about the numerical human data. For example, findings of in vitro mutagens in tobacco smoke can only be interpreted because we know about the association between smoking and lung cancer. Otherwise, such a finding would have no interpretation [55].

Synthesis: a difference in loss function?

We need both hierarchies, the hierarchy of discovery and explanation as well as that of evaluation. Without new discoveries leading to potentially better diagnosis or therapy, what would we do randomised trials on? Conversely, how could we know that a discovery is useful, if not evaluated? The two hierarchies coexist because they serve different purposes.

Still, there is an almost emotional difference. Many researchers enjoy the game of multiplicity of analysis with low priors in observational research. Finding explanations is a puzzle solving process that leads to what Leonardo da Vinci is reputed to have called the noblest pleasure: “*the joy of understanding*”. However, the same researchers can become quite upset at any shortcut or lapse from protocol in randomised trials, because results of such trials are applied to real people and should be true, and not just an interesting idea.

The essential difference between these views may have been well encapsulated in a few lines by Michael Ignatieff, the Harvard academic who turned Canadian politician, about the difference between academics and politicians: “*In academic life, false ideas are merely false and useless ones can be fun to play with. In political life, false ideas can ruin the lives of millions and useless ones can waste precious resources. An intellectual's responsibility for his ideas is to follow their consequences wherever they may lead. A politician's responsibility is to master those consequences…"* [56]. Ignatieff’s academics correspond to the observational

etiologic researchers in the present essay, while his politicians are akin to the researchers that populate the world of randomised trials. The latter want to make decisions with a high degree of certainty, to avoid harm to people.

There is a difference in “loss function” between these two types of activity. R. A. Fisher once explained why significance tests are good for practical decision making – e.g., to decide whether to accept or reject a batch of manufactured goods, because a loss function can be specified: on the one hand lost time and money in making the goods if they are wrongly rejected, on the other hand dissatisfied consumers who turn away if goods are wrongly accepted. Science, Fisher suggested, was different because it is impossible to calculate the loss function of a wrongly held or wrongly rejected explanation. Thus, significance tests would never be useful in science [57]. According to Fisher, the aim of data collection is to quantify predictions from explanations; after seeing the data, reasoning about the explanations continues.

Paraphrasing these ideas, I propose that the loss function of evaluation research - the prototypical randomised trial of drug therapy, concerns real people who are cured or harmed by our acceptance or rejection of a particular therapy. Under equipoise, the data from randomised trials are the best information that we have. We will accept them, always knowing that we can be wrong, but within the limits of “acceptable regret” [58]. Above all, we should not tamper with such data: our delight in exploring new ideas should not be allowed to affect a future patient’s health. Thus, it is right that randomised trials are only started when the odds are favourably large (say, 50-50) and that they are carried out with adequate numbers under tight protocols: the results are applied to people, first of all the people in the trial, and later a much larger number of similar patients. If we get it wrong, in whatever direction, many people may be harmed.

In contrast, the loss function of discovery and explanation cannot be defined equally directly. Aetiologic researchers have a duty to play around with low-probability hypotheses, because these may lead to new insights. Much good can come from going down the wrong alley and detecting why it is wrong, or from playing around with a seemingly useless hypothesis: the real breakthrough might come from that experience. What is lost if we go too far in the wrong direction is time and money for science. That is again inevitable: science makes progress “*in a fitful and meandering way*” as described by Stephen Jay Gould [59]. His words are an echo of

Sir William Osler's in his Harveian oration on the *"Growth of Truth"* in 1906: *"Truth may suffer all the hazards incident to generation and gestation… [and]… all scientific truth is conditioned by the state of knowledge at the time of its announcement"* [60]. Two scientists, working one hundred years apart in totally different fields and totally different circumstances give a similar verdict. Nothing much has changed in the way science progresses, presumably because scientific progress is an activity of human brains, and our brains haven't changed that much.

The idea that science "meanders" when making progress seems difficult to accept. However, the process of sieving out "right" from "wrong", which many persons believe is a matter of having the "right" data, is compounded by the fact to which Osler already alluded: all data analyses are interpretations in the light of particular hypotheses and a particular state of knowledge. All communication about data, like all data collection, is selective and interpretative. This inherent selection and interpretation may lead scientists to stray collectively too far in a wrong alley. Again this is inevitable, as data cannot be collected, nor analysed or communicated, without interpretation [59, 61, 62].

In the present essay, I have deliberately painted contrasting extremes of evaluation vs. discovery and explanation. I have equated the first with randomized trials and the second with observational research - as if all observational research was about discoveries and new ideas. For my purpose of elucidating how the two forms of medical research are different, and to disentangle the several mutual misunderstandings, it was necessary to start with each in its extreme form. Of course, randomised trials can also deal with etiology, as in explanatory trials [63]. Observational research can also be evaluative - particularly if it concerns a much needed replication - and may then also lead to action. Whenever acting, on the basis of whatever type of studies, we should remain aware of potential residual uncertainties. For randomized trials as well as for observational research, the threshold for action will therefore be different according to importance, cost, and balance of potential benefits and adverse effects of the action.

In the end, we will have to live with the fitful and meandering ways of discovery and explanation, and at the same time call for as strict an evaluation as possible before we apply new insights to people. There is no other way forward.

Note added in proof: after acceptance of this essay, I was made aware of a paper about a similar theme: how to bridge basic and applied research, and what the role of epidemiology would be (Swales J. The troublesome search for evidence: three cultures in need of integration. J R Soc Med. 2000 Aug;93(8):402-7)

References

1. Laine C, Horton R, DeAngelis CD, Drazen JM, Frizelle FA, Godlee F, Haug C,Hebert PC, Kotzin S, Marusic A, Sahni P, Schroeder TV, Sox HC, Van der Weyden MB, Verheugt FW. Clinical trial registration--looking back and moving ahead. N Engl J Med 2007;356:2734-6.
2. Trousseau A. Clinique Médicale de l'Hotel-Dieu de Paris. Paris, Librairie J-B Ballière et fils, 1902: Tome I, pp 30-34.
3. Vandenbroucke JP, Koster T, Briet E, Reitsma PH, Bertina RM, Rosendaal FR. Increased risk of venous thrombosis in oral-contraceptive users who are carriers of factor V Leiden mutation. Lancet. 1994;344:1453-7.
4. Vandenbroucke JP, Rosendaal FR, Bertina RM. Factor V Leiden, Oral Contraceptives and Deep Vein Thrombosis. IN: Khoury JM, Little J, Burke W. Human Genome Epidemiology. New York, Oxford Univ Press 2004: 322-332.
5. Vandenbroucke JP, Rosing J, Bloemenkamp KW, Middeldorp S, Helmerhorst FM, Bouma BN, Rosendaal FR. Oral contraceptives and the risk of venous thrombosis. N Engl J Med. 2001;344:1527-35.
6. Vandenbroucke JP, Kroep JR. Bortezomib in multiple myeloma. N Engl J Med. 2005;353:1297-8.
7. Tugwell P, Haynes RB, Sackett DL. Clinical epidemiology: a basic science for clinical medicine. Boston: Little Brown &Co, 1985.

8. Stricker BH, Psaty BM. Detection, verification, and quantification of adverse drug reactions. BMJ. 2004;329:44-7
9. Lee JAH, Vaughan TL, Diehr PH, Haertle RA. The recognition of new kinds of occupational toxicity. In: Castellani, A. Ed. Epidemiology and quantitation of environmental risk in humans from radiation and other agents. New York: Plenum, 1985: 307-37.
10. Glasziou P, Chalmers I, Rawlins M, McCulloch P. When are randomised trials unnecessary? Picking signal from noise. BMJ. 2007;334:349-51.
11. Vandenbroucke JP. In defense of case reports and case series. Ann Intern Med. 2001;134:330-4.
12. Vandenbroucke JP. When are observational studies as credible as randomised trials? Lancet. 2004 May 22;363(9422):1728-31.
13. Schulz KF, Grimes DA. Allocation concealment in randomised trials: defending against deciphering. Lancet. 2002;359:614-8.
14. Miettinen OS. The need for randomization in the study of intended effects. Stat Med. 1983;2:267-71.
15. Vessey and Jick AJE Jick H, Vessey MP. Case-control studies in the evaluation of drug-induced illness. Am J Epidemiol. 1978;107:1-7.
16. Vandenbroucke JP. What is the best evidence for determining harms of medical treatment? CMAJ. 2006;174:645-6.
17. Papanikolaou PN, Christidi GD, Ioannidis JP. Comparison of evidence on harms of medical interventions in randomized and nonrandomized studies. CMAJ. 2006;174:635-41.
18. Little J, Khoury MJ. Mendelian randomisation: a new spin or real progress? Lancet. 2003;362:930-1.

19. Editorial commentary (2007). Identifying unanticipated effects of treatments. The James Lind Library (www.jameslindlibrary.org).

20. Glynn RJ, Schneeweiss S, Wang PS, Levin R, Avorn J. Selective prescribing led to overestimation of the benefits of lipid-lowering drugs. J Clin Epidemiol. 2006;59:819-28.

21. Setoguchi S, Glynn RJ, Avorn J, Mogun H, Schneeweiss S. Statins and the risk of lung, breast, and colorectal cancer in the elderly. Circulation. 2007;115:27-33.

22. Thomsen RW. The lesser known effects of statins: benefits on infectious outcomes may be explained by "healthy user" effect. BMJ. 2006;333:980-1.

23. Majumdar SR, McAlister FA, Eurich DT, Padwal RS, Marrie TJ. Statins and outcomes in patients admitted to hospital with community acquired pneumonia: population based prospective cohort study. BMJ. 2006;333:999.

24. Solomon DH, Schneeweiss S, Glynn RJ, Kiyota Y, Levin R, Mogun H, Avorn J. Relationship between selective cyclooxygenase-2 inhibitors and acute myocardial infarction in older adults. Circulation. 2004;109:2068-73.

25. Rosenbaum PR. Observational studies (2nd Ed). New York: Springer, 2002

26. Wellcome Trust Case Control Consortium. Genome-wide association study of 14,000 cases of seven common diseases and 3,000 shared controls. Nature. 2007;447:661-78.

27. Djulbegovic B, Lacevic M, Cantor A, Fields KK, Bennett CL, Adams JR, Kuderer NM, Lyman GH. The uncertainty principle and industry-sponsored research. Lancet. 2000;356:635-8.

28. Kumar A, Soares H, Wells R, Clarke M, Hozo I, Bleyer A, Reaman G, Chalmers I, Djulbegovic B. Children's Oncology Group. Are experimental treatments for cancer in children superior to established treatments? Observational study of randomised controlled trials by the Children's Oncology Group. BMJ. 2005;331(7528):1295.

29. Rothman KJ. No adjustments are needed for multiple comparisons. Epidemiology. 1990;1:43-6.

30. Greenland S. Commentary: on 'quality in epidemiological research': should we be submitting papers before we have the results and submitting more "hypothesis generating research?" Int J Epidemiol. 2007;36:944-5.

31. Lipton P. Testing hypotheses: prediction and prejudice. Science. 2005;307:219-21.

32. Brush SG. Accommodation or prediction? Science. 2005;308:1409-12

33. Rothwell PM. Treating individuals 2. Subgroup analysis in randomised controlled trials: importance, indications, and interpretation. Lancet. 2005;365:176-86.

34. Joost O, Wilk JB, Cupples LA, Harmon M, Shearman AM, Baldwin CT, O'Connor GT, Myers RH, Gottlieb DJ. Genetic loci influencing lung function: a genome-wide scan in the Framingham Study. Am J Respir Crit Care Med. 2002;165:795-9.

35. Goodman SN. Multiple comparisons, explained. Am J Epidemiol. 1998;147:807-12.

36. Vandenbroucke JP, Bertina RM, Holmes ZR, Spaargaren C, van Krieken JH, Manten B, Reitsma PH. Factor V Leiden and fatal pulmonary embolism. Thromb Haemost. 1998;79:511-6.

37. Thomassen R, Vandenbroucke JP, Rosendaal FR. Antipsychotic drugs and venous thromboembolism. Lancet. 2000;356:252.

38. Walker AM, Lanza LL, Arellano F, Rothman KJ. Mortality in current and former users of clozapine. Epidemiology. 1997;8:671-7.

39. Zornberg GL, Jick H. Antipsychotic drug use and risk of first-time idiopathic venous thromboembolism: a case-control study. Lancet. 2000 Oct 7;356(9237):1219-23.

40. Moonesinghe R, Khoury MJ, Janssens AC. Most published research findings are false-but a little replication goes a long way. PLoS Med. 2007;4:e28.

41. Khoury MJ, Little J, Gwinn M, Ioannidis JP. On the synthesis and interpretation of consistent but weak gene-disease associations in the era of genome-wide association studies. Int J Epidemiol. 2007;36:439-45.

42. Vandenbroucke JP, von Elm E, Altman DG, Gotzsche PC, Mulrow CD, Pocock SJ, Poole C, Schlesselman JJ, Egger M for the STROBE initiative. Strengthening the Reporting of Observational Studies in Epidemiology (STROBE): Explanation and Elaboration. PLoS Medicine 2007;10:e297.

43. Maclure M. Demonstration of deductive meta-analysis: ethanol intake and risk of myocardial infarction. Epidemiol Rev. 1993;15:328-51.

44. Ebrahim S. Cohorts, infants and children. Int. J. Epidemiol. 2004;33:1165-1166

45. Ioannidis JP. Why most published research findings are false. PLoS Med. 2005;2:e124.

46. Goodman S, Greenland S. Why most published research findings are false: problems in the analysis. PLoS Med. 2007;4:e168.

47. Cornfield J. Statistical relationships and proof in medicine. American Statistician 1954;8:19-21. Reprinted in: Greenland S. Evolution of epidemiologic ideas; annotated readings on concepts and methods. Epidemiology Resources, Inc. 1987: 10-13.

48. Doll R. Cohort studies: history of the method. I. Prospective cohort studies. Soz Praventivmed. 2001;46:75-86.

49. Doll R, Hill AB. Smoking and carcinoma of the lung: preliminary report. Br Med J 1950; ii: 739–48

50. Rosendaal FR. Bridging case-control studies and randomized trials. Curr Control Trials Cardiovasc Med. 2001;2:109-110.

51. Miettinen O. Estimability and estimation in case-referent studies. Am J Epidemiol. 1976;103:226-35.

52. Mayes LC, Horwitz RI, Feinstein AR. A collection of 56 topics with contradictory results in case-control research. Int J Epidemiol. 1988;17:680-5.
53. Schulz KF, Grimes DA. Case-control studies: research in reverse. Lancet. 2002;359:431-4.
54. Cornfield J, Haenszel W, Hammond EC, Lilienfeld AM, Shimkin MB, Wynder EL. Smoking and lung cancer: recent evidence and a discussion of some questions. J Natl Cancer Inst 1959:22:173-203.
55. Vandenbroucke JP. 175th anniversary lecture. Medical journals and the shaping of medical knowledge. Lancet 1998;352:2001-6.
56. Ignatieff M. Getting Iraq wrong. New York Times Magazine, August 5, 2007 (third paragraph).
57. Fisher RA. Statistical methods and scientific inference (3rd Ed). New York: Hafner Press, 1973
58. Djulbegovic B, Hozo I. When should potentially false research findings be considered acceptable? PLoS Med. 2007;4:e26.
59. SJ Gould, Pathways of discovery: deconstructing the “science wars” by reconstructing an old mold. Science 2000:287:253–261.
60. W Osler, Harveian oration. The growth of truth, as illustrated in the discovery of the circulation of the blood. BMJ 1906;ii:1077–1084.
61. Vandenbroucke JP, de Craen AJ. Alternative medicine: a "mirror image" for scientific reasoning in conventional medicine. Ann Intern Med. 2001;135:507-13.
62. McAllister JW. Algorithmic randomness in empirical data. Stud Hist Phil Sci 2003;34:633-46.
63. Schwartz D, Lellouch J. Explanatory and pragmatic attitudes in clinical trials. J Chron Dis 1967; 20: 637-648.
